# Sexual Function and Depressive Symptoms in Metformin-Treated Women with Drug-Induced Hyperprolactinemia and Different Vitamin D Status: A Pilot Study

**DOI:** 10.3390/pharmaceutics18030376

**Published:** 2026-03-18

**Authors:** Robert Krysiak, Witold Szkróbka, Karolina Kowalcze, Bogusław Okopień

**Affiliations:** 1 Department of Internal Medicine and Clinical Pharmacology, Medical University of Silesia, Medyków 18, 40-752 Katowice, Poland; rkrysiak@sum.edu.pl (R.K.); bokopien@sum.edu.pl (B.O.); 2 Department of Pediatrics in Bytom, Faculty of Health Sciences in Katowice, Medical University of Silesia, Stefana Batorego 15, 41-902 Bytom, Poland; kkowalcze@sum.edu.pl; 3 Department of Pathophysiology, Faculty of Medicine, Academy of Silesia, Rolna 43, 40-555 Katowice, Poland

**Keywords:** antipsychotics, depressive symptoms, female sexual function, prolactin excess, premenopausal women, sex hormones

## Abstract

**Background:** Elevated prolactin levels are associated with disturbances in female sexual function. While long-term therapy with dopamine agonists has been shown to improve these disturbances, the therapeutic benefits appear to be reduced in the presence of vitamin D deficiency or insufficiency. Therefore, the present study aimed to examine whether vitamin D status modulates the effects of metformin—a medication with less pronounced prolactin-lowering properties—on sexual function and depressive symptoms. **Methods:** The study cohort comprised three groups of reproductive-age women with drug-induced hyperprolactinemia and prediabetes, matched for age, glycated hemoglobin, and prolactin concentrations. Group I included 25 women with normal vitamin D status who were not receiving vitamin D supplementation. Group II consisted of 25 women with vitamin D deficiency or insufficiency that was adequately corrected through supplementation, while group III included 25 women with untreated vitamin D deficiency or insufficiency. All participants received metformin throughout the six-month study period. Female sexual function and depressive symptoms were assessed before and after metformin therapy using the Female Sexual Function Index (FSFI) and the Beck Depression Inventory-II (BDI-II), respectively. Additional outcome measures included plasma 25-hydroxyvitamin D, fasting plasma glucose, glycated hemoglobin (HbA_1c_), the homeostatic model assessment of insulin resistance (HOMA-IR), prolactin, gonadotropins, and sex hormones. **Results:** Improvements in glucose homeostasis were observed across all groups; however, these changes were more pronounced in groups I and II than in group III. Reductions in prolactin concentrations (total and monomeric), accompanied by increases in gonadotropins, estradiol, and testosterone, were observed exclusively in women with normal vitamin D status. In groups I and II, metformin therapy resulted in significant improvements in total FSFI scores as well as in all individual domain scores. In contrast, in group III, the effects of metformin were limited to increases in the domain scores for lubrication and sexual satisfaction. Improvements in sexual function were positively associated with baseline 25-hydroxyvitamin D levels, reductions in prolactin concentrations, and, to a lesser extent, treatment-related changes in HbA_1c_ and increases in testosterone. A treatment-induced reduction in total BDI-II scores was observed only among women with normal vitamin D status. **Conclusions:** Low vitamin D status diminishes the beneficial effects of metformin on sexual function and depressive symptoms in reproductive-age women with iatrogenic hyperprolactinemia.

## 1. Introduction

In addition to causing secondary amenorrhea or oligomenorrhea, galactorrhea, and infertility [1,2], elevated prolactin concentrations have been shown to adversely affect all domains of female sexual function, with the magnitude of impairment correlating with the severity of hyperprolactinemia [3,4]. Among women with pituitary or hypothalamic tumors, reduced libido was reported 2.5 times more frequently than in age- and disease-matched individuals whose prolactin levels were within the reference range [5]. Furthermore, in women with polycystic ovary syndrome, prolactin concentrations were inversely associated with both the frequency and quality of orgasm [6]. Lastly, sexual function in women with hyperprolactinemia has been shown to improve following treatment with dopamine agonists—the pharmacological therapy of choice for this condition—concomitant with reductions in prolactin levels [7,8].

Another endogenous compound shown to influence the female sexual response is vitamin D. In infertile Turkish women, both mean total FSFI scores and scores across all sexual domains were lower in those with vitamin D deficiency compared with peers whose 25OHD levels exceeded 50 nmol/L; moreover, scores were lower in women with vitamin D deficiency than in those with insufficiency [9]. Another study conducted in Turkey demonstrated that 25OHD levels were significantly lower in patients with female sexual dysfunction (15.9 ± 8.4 nmol/L) than in women without this condition (26.3 ± 11.7 nmol/L) [10]. Among young Polish women, sexual dysfunction was also observed, though it was less pronounced. Women with vitamin D deficiency exhibited lower overall FSFI scores and reduced scores in the domains of sexual desire, orgasm, and satisfaction, whereas women with vitamin D insufficiency showed reduced scores only in the desire domain [11]. Vitamin D status may also modulate the sexual response to dopamine agonists, potentially accounting for differences in their effects. In the case of bromocriptine, improvement in sexual functioning—less pronounced than that observed with cabergoline—was noted in a population that had not been selected based on vitamin D status, although participants lived in a region where vitamin D deficiency is common among women of reproductive age [7]. In contrast, for cabergoline, improvement in sexual functioning was clearly associated with vitamin D status and appeared to be most pronounced in individuals with adequate vitamin D levels [8].

Importantly, adequate vitamin D supplementation appears to confer beneficial effects on sexual health [12,13,14,15]. Intramuscular administration of exogenous vitamin D (50,000 IU per week for 12 weeks) improved all aspects of sexual function in women with polycystic ovary syndrome and vitamin D deficiency, with no additional benefit observed in those concurrently receiving oral contraceptive therapy [12]. Similarly, intramuscular administration of 300,000 IU at baseline and again after four weeks in women with sexual dysfunction and 25OHD levels below 75 nmol/L resulted in increases in both overall FSFI scores and domain-specific scores across all FSFI subscales [13]. Administration of 300,000 IU intramuscularly followed by 50,000 IU orally once weekly for four weeks also improved overall FSFI scores in young women with female sexual dysfunction [14]. Finally, a low-dose regimen of vitamin D (600 IU daily for six weeks) led to resolution of sexual dysfunction in reproductive-aged women with polycystic ovary syndrome, irrespective of concurrent hormonal contraceptive use [15]. Unfortunately, the evaluation of vitamin D supplementation on sexual function has been limited to cases of isolated vitamin D deficiency or coexisting polycystic ovary syndrome.

Hyperprolactinemia induced by antipsychotic medications represents one of the most challenging forms of this condition to manage. Strategies such as reducing the dose or discontinuing the offending antipsychotic, switching to prolactin-sparing agents, implementing adjunctive aripiprazole therapy, or administering dopamine agonists may exacerbate psychotic symptoms, precipitate relapse of the primary psychiatric disorder, or increase the risk of adverse effects [16,17]. Evidence from double-blind, randomized, placebo-controlled trials and meta-analyses of randomized controlled studies indicates that chronic administration of metformin effectively reduces prolactin levels in patients with antipsychotic-induced hyperprolactinemia, whereas no significant effect is observed in placebo-treated individuals [18,19,20,21]. The prolactin-lowering effect of metformin, likely mediated through inhibition of the secretory activity of overactive lactotrophs, appears to be more pronounced in women than in men and is dose-dependent, with greater reductions observed at higher doses [19,22]. Importantly, metformin does not reduce prolactin levels in individuals whose concentrations remain within the reference range, suggesting that its use is unlikely to induce clinically relevant prolactin deficiency [23]. An additional rationale for metformin administration is its favorable impact on carbohydrate metabolism, which is particularly relevant given that both psychiatric disorders and antipsychotic therapy may contribute to hyperglycemia, insulin resistance, and an increased risk of type 2 diabetes mellitus or prediabetic states [24,25,26]. Women exhibit increased susceptibility to metabolic complications associated with antipsychotic use [27].

To the best of our knowledge, no previous study has examined the impact of metformin on female sexual function in women with hyperprolactinemia. Only two studies have explored the association between sexual health and metformin use, yielding conflicting results. In women with type 2 diabetes or prediabetes, metformin was reported to normalize overall sexual functioning as well as all individual domains [28]. In contrast, another study found that the prevalence of metformin use among women with diabetes was higher in those with sexual dysfunction than in those without this complication [29]. However, this latter observation may represent an epiphenomenon attributable to differences in diabetes severity between the two groups. To further elucidate the relationship between metformin use and female sexual response, the present study aimed to investigate whether metformin affects sexual function in women with antipsychotic-induced hyperprolactinemia, whether any such effect is accompanied by changes in depressive symptoms, and whether these potential effects are influenced by patients’ vitamin D status.

## 2. Materials and Methods

This single-center, prospective, matched outpatient cohort study was conducted in accordance with the principles of the Declaration of Helsinki and was approved by the institutional review board. Written informed consent was obtained from all participants prior to enrollment.

### 2.1. Study Population

Participants were recruited from premenopausal women aged 18–50 years with previously untreated prediabetes, diagnosed according to standard criteria [30]. Due to an inadequate response to at least 12 weeks of lifestyle interventions, all participants were considered candidates for metformin therapy. Inclusion required mild to moderate hyperprolactinemia (prolactin levels between 40 and 80 ng/mL [850–1700 mU/L] on two separate occasions at least six weeks apart) induced by chronic antipsychotic treatment, with stable medication doses for a minimum of 12 weeks. Patients were required to be receiving either a first- or second-generation antipsychotic with a documented propensity to induce hyperprolactinemia. Those receiving atypical antipsychotic medications with a low potential to elevate prolactin levels were excluded due to the risk of an alternative etiology of hyperprolactinemia. The severity of schizophrenia symptoms was measured using the six-item Positive and Negative Syndrome Scale (PANNS-6). Enrolled participants were assigned to one of three groups, each comprising 25 women. Groups I and II included women with normal vitamin D status (25-hydroxyvitamin D [25OHD] levels between 75 and 150 nmol/L [30–60 ng/mL]) [31], with group II receiving ongoing vitamin D supplementation (25–100 μg [1000–4000 IU] daily) for at least 12 weeks due to prior deficiency or insufficiency. Group I did not receive supplementation. Group III consisted of women with 25OHD levels of 25–75 nmol/L (10–30 ng/mL), indicative of deficiency or insufficiency, who were neither receiving nor wished to receive vitamin D supplementation. For ethical reasons, individuals with 25OHD levels below 25 nmol/L (10 ng/mL) were excluded from group III. This threshold was established because 25OHD concentrations below this level indicate severe vitamin D deficiency, which is associated with an increased risk of osteomalacia, falls, fractures, and myopathy [32,33]. However, a prior history of severe vitamin D deficiency did not preclude enrollment in group II, provided that participants’ vitamin D status had been normalized through exogenous supplementation at the time of study initiation. Additionally, to reduce the risk of secondary hypogonadism, patients with prolactin levels above 80 ng/mL (1700 mU/L) were excluded from participation [34]. Although 20 participants per group would have been sufficient to detect a 15% difference in total FSFI score—the study’s primary endpoint—with 80% statistical power and a 0.05 significance threshold, group sizes were increased to account for potential dropouts and loss to follow-up. Study groups were drawn from larger pools of eligible candidates using a computerized matching system to ensure comparability in age, glycated hemoglobin (HbA_1c_), and prolactin levels. To mitigate the influence of seasonal fluctuations in vitamin D status [35], recruitment was conducted throughout the year, with roughly equal numbers of participants enrolled in each season.

Participants were further excluded if they exhibited any of the following: diabetes; hyperprolactinemia of other or mixed origin; additional endocrine disorders; cardiovascular, renal, or hepatic disease; anemia or malabsorption syndromes; sexual inactivity; pregnancy, lactation, or plans to conceive; congenital or acquired reproductive system anomalies; prior urogynaecological surgeries that could affect sexual function; or ongoing hormonal contraception or other pharmacological treatments, except for antipsychotics and, in group II, vitamin D supplementation.

### 2.2. Study Design

All participants received immediate-release metformin orally, taken with or immediately after meals. The dose was gradually titrated every 4–7 days based on individual tolerance, from an initial 500 mg twice daily to a target dose of 850–1000 mg three times daily, which was maintained for six months. Participants in group II also continued vitamin D supplementation at their pre-study dose (25–100 μg daily). Treatment adherence was monitored at each visit, conducted every two months, using pill counts and participant interviews. Concomitant medications—including nonsteroidal anti-inflammatory drugs, acetaminophen, hypnotics, antidiarrheals, laxatives, antitussives, and antimicrobials—were allowed only if administered for ≤10 days and discontinued at least eight weeks before study completion. Participants were instructed to maintain their usual lifestyle throughout the study. Dietary vitamin D intake over the two weeks preceding the study was assessed using participant self-reports, documenting the frequency and portion size of the 20 most commonly consumed Polish dishes. Daily intake for each item was calculated by multiplying the portion size by its vitamin D content, and the results were summed to estimate total dietary vitamin D intake. Nutrient composition data were obtained from the official food composition tables of the Polish National Food and Nutrition Institute [36]. Dietary vitamin D intake was assessed at each follow-up visit throughout the study using a similar methodology. Each observation period spanned seven days and corresponded to every fourth week. The average dietary vitamin D intake during the study was calculated as the mean of these measurements. Total vitamin D intake in group II was calculated by combining dietary intake with supplemental vitamin D from tablets or capsules.

### 2.3. Laboratory Assays

Venous blood samples were obtained from all participants on the first and last day of the study. In menstruating women, samples were collected between days 2 and 5 of the menstrual cycle. Prior to venipuncture, participants remained seated and rested for at least 30 min, with all blood draws performed consistently between 7:00 and 9:00 a.m. Prolactin concentrations were measured from three samples collected at 20-min intervals [37]. Additionally, monomeric prolactin levels were measured in samples from 18 patients per group using the polyethylene glycol precipitation method [19]. All assays were performed in duplicate, and the mean value was used to enhance measurement reliability. Laboratory personnel were blinded to participant group assignments. Plasma glucose and whole-blood HbA_1c_ were determined using an automated analyzer (COBAS Integra, Roche Diagnostics, Basel, Switzerland). Plasma concentrations of 25OHD, insulin, prolactin, gonadotropins, estradiol, testosterone and dehydroepiandrosterone sulfate (DHEA-S) and were quantified via direct chemiluminescence using acridinium ester technology (ADVIA Centaur XP Immunoassay System, Siemens Healthcare Diagnostics, Munich, Germany). Insulin sensitivity was estimated using the homeostasis model assessment (HOMA-IR), calculated as fasting glucose (mmol/L) multiplied by fasting insulin (mU/L) divided by 22.5.

### 2.4. Questionnaires

Immediately following blood collection, participants were asked to complete three questionnaires designed to assess sociodemographic characteristics, sexual functioning (using the Female Sexual Function Index [FSFI]), and the presence and severity of depressive symptoms (using the Beck Depression Inventory, Second Edition [BDI-II]). All questionnaires were completed independently in a private room, without assistance or interference from the research staff. During this phase of the visit, both participants and investigators remained blinded to the biochemical test results.

The FSFI is a 19-item self-administered questionnaire developed to assess multiple dimensions of female sexual function. It evaluates six domains over the preceding four weeks: desire (items 1–2), arousal (items 3–6), lubrication (items 7–10), orgasm (items 11–13), satisfaction (items 14–16), and pain (items 17–19) [38]. Responses are scored on a scale ranging from 0 to 5 or 1 to 5, with a score of 0 indicating no sexual activity during the assessed period [39]. The total FSFI score is calculated by summing domain scores, each multiplied by a specific domain factor (0.6 for desire; 0.3 for arousal and lubrication; and 0.4 for orgasm, satisfaction, and pain). Lower scores on either the total scale or individual domains indicate poorer sexual function. A total score of 26.55 or lower is considered indicative of female sexual dysfunction [38,39].

The Beck Depression Inventory–II (BDI-II) is a 21-item self-administered instrument used to assess the presence and severity of depressive symptoms [40]. The questionnaire covers emotional, cognitive, motivational, and somatic aspects of depression and is based on diagnostic criteria outlined in the Diagnostic and Statistical Manual of Mental Disorders (DSM-IV), enabling standardized evaluation of symptom severity [41]. Participants are instructed to select the statement that best reflects their experiences over the previous two weeks, including the day of assessment. Each item is rated on a 4-point scale ranging from 0 to 3, yielding a total score between 0 and 63. Higher total scores indicate greater severity of depressive symptoms. Depression severity is classified as minimal or absent (0–13), mild (14–19), moderate (20–28), or severe (≥29) [40].

### 2.5. Statistical Analysis

All outcome variables were log-transformed to satisfy the assumptions of normality and homogeneity of variance. Between-group comparisons at each time point were performed using one-way analysis of variance followed by Bonferroni’s post hoc multiple comparison test. Within-group changes in variable means were evaluated using paired Student’s *t*-tests. Categorical variables were analyzed using the chi-square test. Associations between continuous variables were assessed using Pearson’s correlation coefficient (r). In addition, a Sobel test was conducted to determine whether sex hormones had significant mediating effects on the association between the effects of metformin on prolactin levels and on sexual function. *p*-values adjusted for multiple comparisons were considered statistically significant at <0.05.

## 3. Results

### 3.1. Demographic and Clinical Characteristics of the Study Groups

At study entry, the groups did not differ in age, antipsychotic treatment, distribution of prediabetes subtypes, body mass index, smoking status, causes of hyperprolactinemia, physical activity, educational level, occupational status and type of work, number of sexual partners, marital history (number and duration of marriages), number of deliveries and miscarriages, or blood pressure. Significant between-group differences were observed in total daily vitamin D intake. Dietary vitamin D intake from food sources (excluding tablets or capsules) was higher in group I than in the other two groups. Mean total vitamin D intake (from both dietary sources and supplements) was highest in group II, followed by group I, and lowest in group III. Except for lower plasma 25OHD levels in group III, no between-group differences were detected in the measured outcome variables, including fasting glucose, HOMA-IR, HbA_1c_, prolactin (total and monomeric), LH, FSH, estradiol, testosterone, DHEA-S, PANSS-6 score, overall FSFI score, and total BDI-II score (Table 1).

Group I received phenothiazines (n = 4), haloperidol (n = 5), sulpiride (n = 2), flupenthixol (n = 2), risperidone (n = 10), and amisulpride (n = 2). Group II received phenothiazines (n = 5), haloperidol (n = 3), sulpiride (n = 2), flupenthixol (n = 3), risperidone (n = 11), and amisulpride (n = 1). Group III received phenothiazines (n = 3), haloperidol (n = 4), sulpiride (n = 3), flupenthixol (n = 2), risperidone (n = 12), and amisulpride (n = 1). Average and individual daily antipsychotic doses are shown in Table 2.

### 3.2. Progression of Participants Through the Study

**Figure 1:**
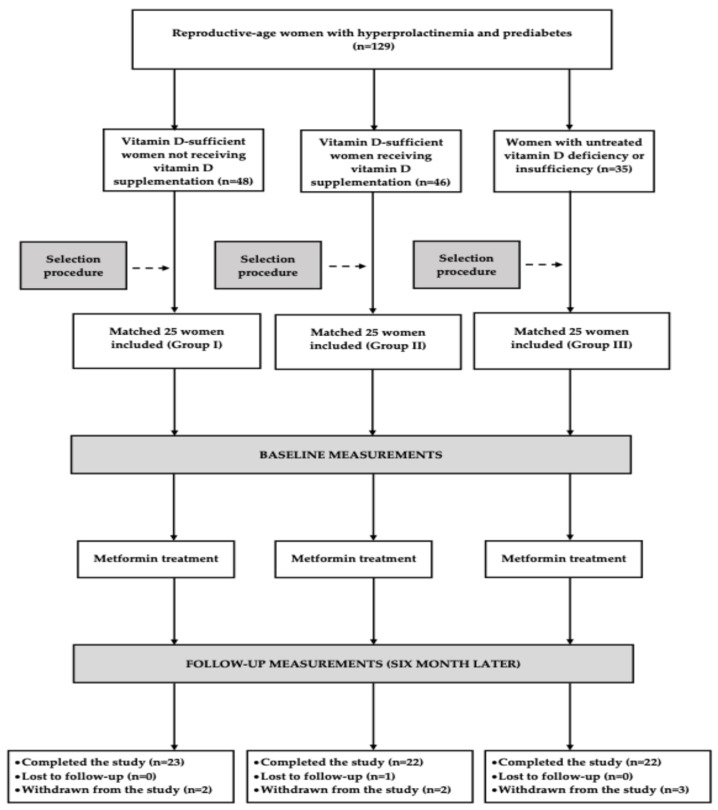
Enrollment and progression of participants through the study.

Of the 75 women initially enrolled in the study, 67 (89%) completed the protocol: 23 in group I and 22 in groups II and III. The most common reason for early withdrawal, affecting four participants (two from group II, one from group I, and one from group III), was the need to modify antipsychotic treatment. Two participants (one each from groups I and III) discontinued participation due to metformin-related adverse effects. One participant from group III withdrew due to inadequate adherence to therapeutic recommendations, while another participant (from group II) ceased attending study visits without providing a reason. Post hoc analysis indicated that the number of participants who completed the full study protocol was sufficient to support statistical analyses. Figure 1 illustrates the progression of participants through the study.

The average dietary vitamin D intake during the study was 18.0 ± 7.5 µg in group I, 9.5 ± 3.8 µg in group II, and 9.1 ± 2.8 µg in group III. Intake was higher in group I than in the other two groups but did not differ from pre-study intake. When vitamin D from tablets and capsules was included, total vitamin D intake in group II was 48.6 ± 17.6 µg, which did not differ from pre-study intake in this group, but was higher than intake during the study in the other groups.

At the end of the study, PANSS-6 scores were 10.4 ± 2.4, 9.7 ± 2.0, and 10.0 ± 2.1 for groups I, II, and III, respectively, with no significant differences observed between the groups or relative to baseline values within each group.

### 3.3. Biochemical Variables

**Figure 2:**
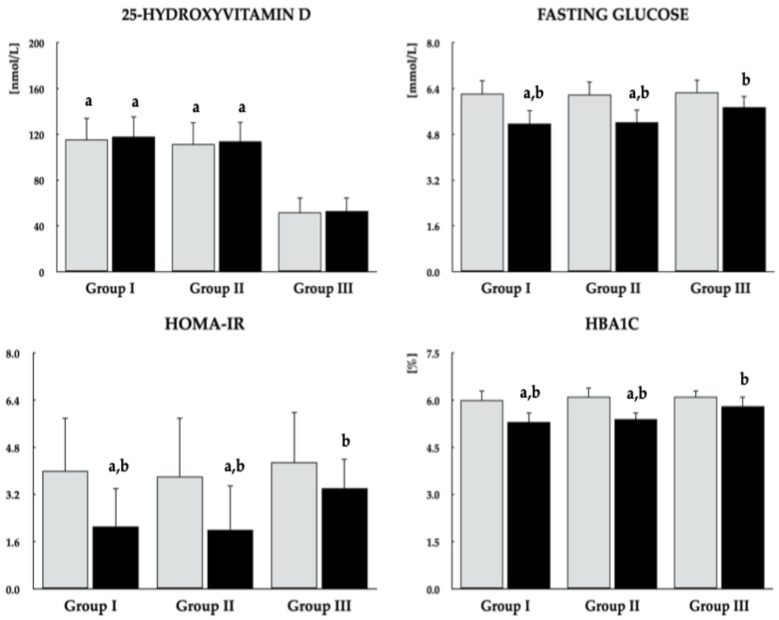
Effect of metformin on 25OHD concentrations and glucose homeostasis in reproductive-age women with antipsychotic-induced hyperprolactinemia stratified by vitamin D status. Group I: vitamin D-sufficient women not receiving vitamin D supplementation; Group II: vitamin D-sufficient women receiving vitamin D supplementation due to previously documented vitamin D deficiency or insufficiency; Group III: women with untreated vitamin D deficiency or insufficiency. Values are reported as mean ± standard deviation. Gray bars represent values at baseline; black bars represent values at study completion. ^a^ *p* < 0.05 vs. the corresponding value in group III. ^b^ *p* < 0.05 vs. baseline values within the same group.

**Figure 3:**
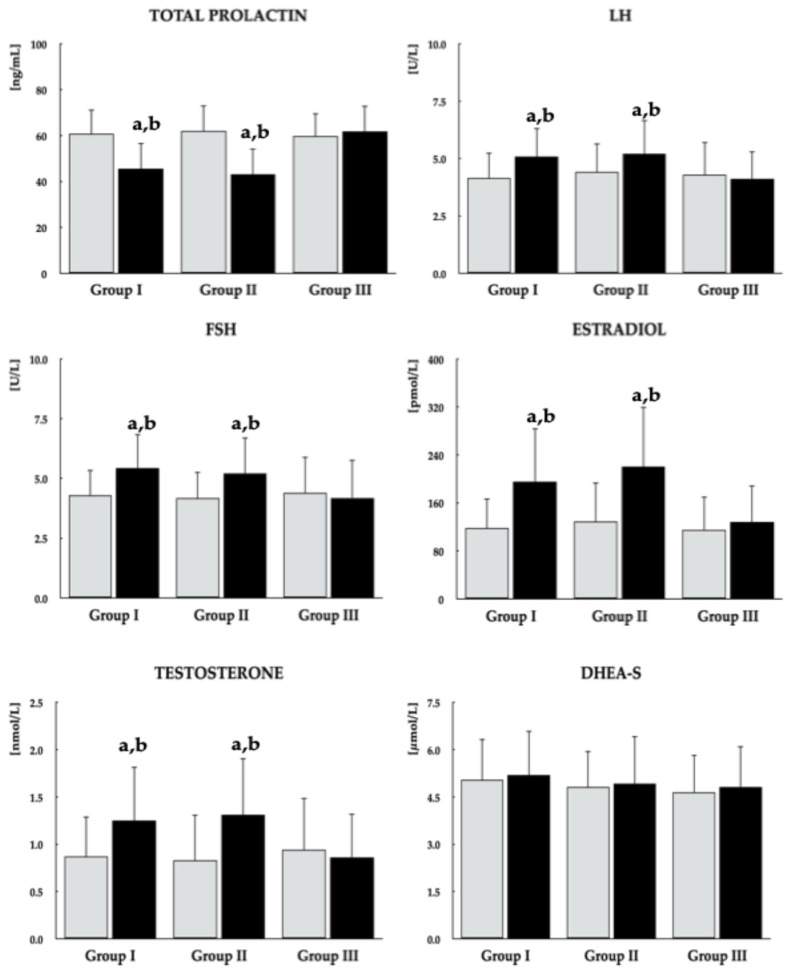
Effect of metformin on hormone concentrations in reproductive-age women with antipsychotic-induced hyperprolactinemia stratified by vitamin D status. Group I: vitamin D-sufficient women not receiving vitamin D supplementation; Group II: vitamin D-sufficient women receiving vitamin D supplementation due to previously documented vitamin D deficiency or insufficiency; Group III: women with untreated vitamin D deficiency or insufficiency. Values are reported as mean ± standard deviation. Gray bars represent values at baseline; black bars represent values at study completion. ^a^ *p* < 0.05 vs. the corresponding value in group III. ^b^ *p* < 0.05 vs. baseline values within the same group.

**Figure 4:**
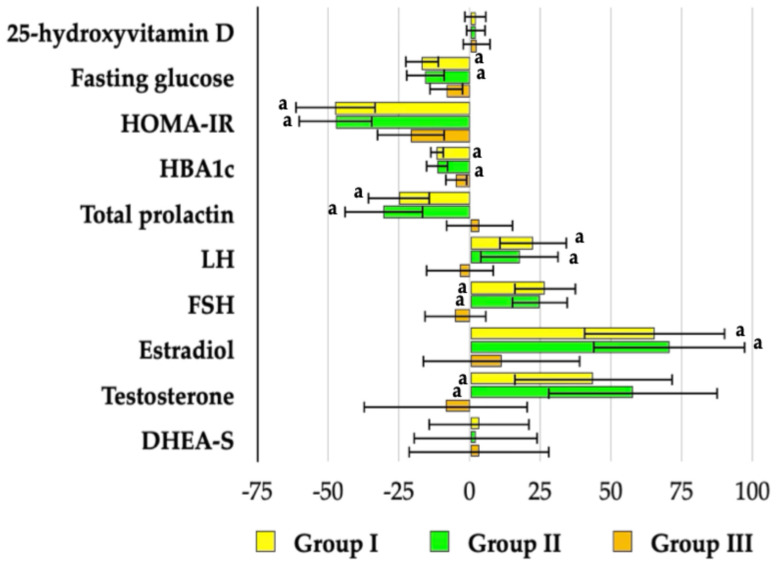
Percentage changes from baseline in biochemical markers in reproductive-age women with antipsychotic-induced hyperprolactinemia stratified by vitamin D status. Group I: vitamin D-sufficient women not receiving vitamin D supplementation; Group II: vitamin D-sufficient women receiving vitamin D supplementation due to previously documented vitamin D deficiency or insufficiency; Group III: women with untreated vitamin D deficiency or insufficiency. Values are reported as mean ± standard deviation. ^a^ *p* < 0.05—percentage changes from baseline were more pronounced than in Group III.

Metformin had no effect on 25OHD and DHEA-S concentrations in any patient group. Although the drug significantly reduced fasting glucose, HOMA-IR, and HbA_1c_ across all study groups, these effects were more pronounced in groups I and II, which also exhibited lower post-treatment values compared with group III. Reductions in total prolactin and gonadotropin levels, along with increases in estradiol and testosterone, were observed only in groups I and II. No significant differences were observed in post-treatment levels of the assessed biochemical outcomes between the two groups of vitamin D-sufficient women (Figure 2, Figure 3 and Figure 4). In groups I and II, mean post-treatment monomeric prolactin concentrations in samples from 18 patients per group (43.0 ± 14.1 ng/mL and 40.2 ± 14.5 ng/mL, respectively) were lower than baseline values (*p* = 0.0014 and *p* = 0.0001), whereas post-treatment levels in group III (58.4 ± 12.9 ng/mL) did not differ from baseline (*p* = 0.7543). Post-treatment monomeric prolactin levels were higher in group III than in groups I (*p* = 0.0016) and II (*p* = 0.0003).

### 3.4. Sexual Functioning

**Figure 5:**
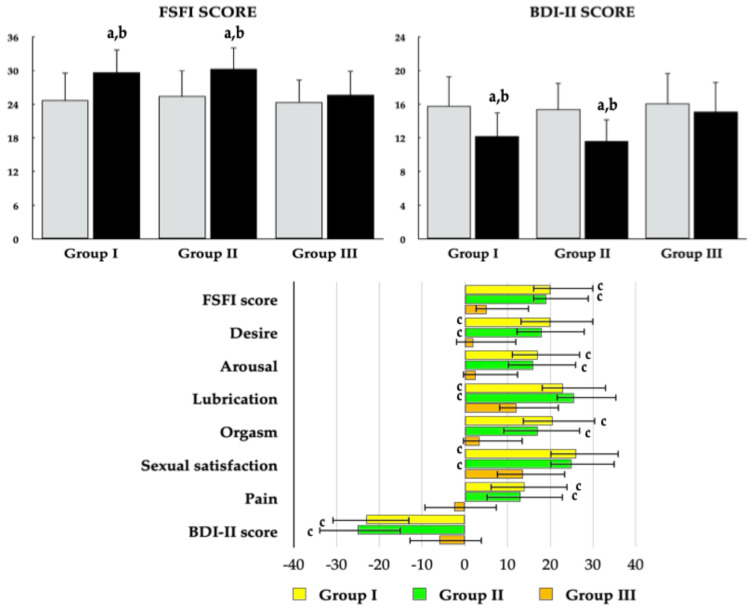
Effect of metformin on total FSFI and BDI -II score and percentage changes from baseline in sexual functioning and depressive symptoms in reproductive-age women with antipsychotic-induced hyperprolactinemia stratified by vitamin D status. Group I: vitamin D-sufficient women not receiving vitamin D supplementation; Group II: vitamin D-sufficient women receiving vitamin D supplementation due to previously documented vitamin D deficiency or insufficiency; Group III: women with untreated vitamin D deficiency or insufficiency. Values are reported as mean ± standard deviation. Gray bars represent values at baseline; black bars represent values at study completion. ^a^ *p* < 0.05 vs. the corresponding value in group III. ^b^ *p* < 0.05 vs. baseline values within the same group. ^c^ *p* < 0.05—percentage changes from baseline were more pronounced than in Group III.

**Figure 6:**
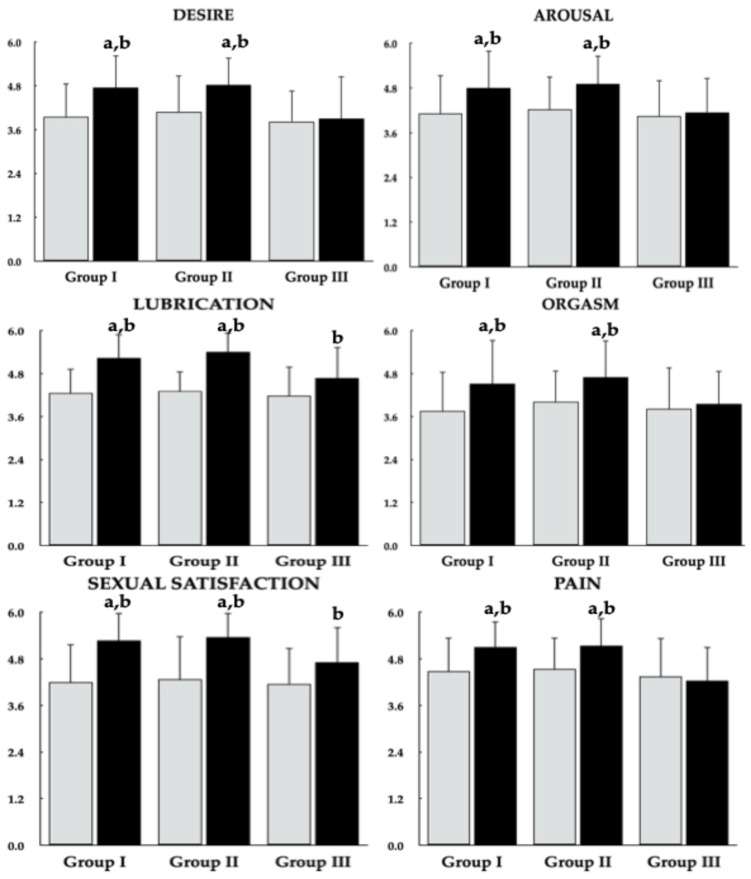
Effect of metformin on components of sexual function in reproductive-age women with antipsychotic-induced hyperprolactinemia stratified by vitamin D status. Group I: vitamin D-sufficient women not receiving vitamin D supplementation; Group II: vitamin D-sufficient women receiving vitamin D supplementation due to previously documented vitamin D deficiency or insufficiency; Group III: women with untreated vitamin D deficiency or insufficiency. Values are reported as mean ± standard deviation. Gray bars represent values at baseline; black bars represent values at study completion. ^a^ *p* < 0.05 vs. the corresponding value in group III. ^b^ *p* < 0.05 vs. baseline values within the same group. ^c^ *p* < 0.05—percentage changes from baseline were more pronounced than in Group III.

Metformin administration resulted in an increase in the total FSFI score and all domain scores in Groups I and II. In Group III, metformin increased the lubrication and sexual satisfaction domain scores but had no significant effect on the total FSFI score or the domains of desire, arousal, orgasm, and pain. Percentage changes in the total FSFI score and all domain scores were more pronounced in vitamin D–sufficient women than in women with untreated vitamin D deficiency or insufficiency. A reduction in the proportion of patients with an FSFI score ≤ 26.55 was observed exclusively in groups I and II (Figure 5 and Figure 6, Table 3).

### 3.5. Depressive Symptoms

Metformin administration led to a reduction in the total BDI-II score in groups I and II. Similarly, only in these groups did the drug decrease the proportion of patients with total and mild depressive symptoms. The effects of metformin on both the BDI-II score and the proportion of affected patients were more pronounced in vitamin D–sufficient women than in those with untreated vitamin D deficiency or insufficiency (Figure 5, Table 3).

### 3.6. Correlations and Mediation Analysis

At baseline, weak positive correlations were observed between the total FSFI score, all domain scores, and baseline 25OHD levels in group III (r = 0.25 to 0.35; *p* < 0.05). The effect of metformin on prolactin concentrations in groups I and II was positively correlated with baseline prolactin levels (r = 0.55, *p* < 0.001 and r = 0.60, *p* < 0.001 for total prolactin, and r = 0.60, *p* < 0.001 and r = 0.62, *p* < 0.001 for monomeric prolactin, respectively) as well as with changes in LH concentrations (r = 0.59, *p* < 0.001 and r = 0.56, *p* < 0.001 for total prolactin, and r = 0.57, *p* < 0.001 and r = 0.58, *p* < 0.001 for monomeric prolactin, respectively). Changes in LH concentrations were, in turn, positively correlated with changes in testosterone levels (r = 0.51, *p* < 0.001 and r = 0.49, *p* < 0.001, respectively). The effects of metformin on the total FSFI score and all domain scores were positively correlated with changes in total and monomeric prolactin concentrations. Changes in sexual desire and arousal were positively correlated with changes in testosterone levels, with these associations being stronger than those observed with prolactin. Improvements in lubrication and orgasm were positively correlated with changes in HbA_1c_ (Table 4). In group III, but not in groups I or II, positive correlations were observed between the effects of metformin on all assessed sexual outcomes and baseline 25OHD concentrations (Table 5). Finally, the effect of metformin on the total BDI-II score was positively correlated with metformin-induced changes in the total FSFI score and all domain scores (Table 6). The effect of metformin on FSFI and BDI-II scores did not correlate with baseline PANSS-6 scores or with the effect of metformin on PANSS-6 scores.

In the Sobel test, only testosterone was a significant mediator for the associations between the impact of metformin on prolactin levels and its impact on desire (z-score between 2.28 [*p* = 0.0255] and 2.96 [0.0008]) and arousal (z-score between 2.08 [*p* = 0.0455] and 2.80 [0.0125]). Other hormones did not have significant mediating effects on any aspect of sexual functioning.

## 4. Discussion

The study demonstrated that six months of high-dose metformin treatment in young women with drug-induced hyperprolactinemia and normal vitamin D status resulted in a significant reduction in prolactin levels, which correlated with baseline concentrations. The decrease in total prolactin resulted from a reduction in the monomeric form of this hormone. However, at the end of the study, prolactin levels remained above the reference range, supporting the preference for other therapeutic options in the management of hyperprolactinemia, particularly dopaminergic agents. Metformin may, however, serve as an alternative in cases of intolerance or contraindications to these medications and can also be considered as part of combination therapy, allowing for a reduction in the dose of a dopamine agonist or aripiprazole. Furthermore, the results suggest that in patients requiring pharmacologic management of hyperglycemia, the presence of concomitant hyperprolactinemia may justify selecting metformin among the available hypoglycemic treatment options.

Another finding worth noting was the absence of a reduction in total and monomeric prolactin levels in cases of uncompensated vitamin D deficiency. Similarly, increases in LH and testosterone were observed only in women with normal vitamin D status. The positive correlations between changes in prolactin and LH, as well as between LH and sex hormones, indicate that the increase in activity of the hypothalamic–pituitary–gonadal axis was secondary to the reduction in prolactin levels. Moreover, the magnitude of the drug’s effects on glucose levels, HbA_1c_, and HOMA-IR was greater in individuals with adequate vitamin D status. Collectively, these observations emphasize the importance of assessing 25OHD levels in patients with antipsychotic-induced hyperprolactinemia as a predictive marker of metformin efficacy.

Among the evaluated patients, no differences were observed between study groups in baseline PANSS-6 scores. No changes in this parameter occurred during treatment, nor were any correlations found between PANSS-6 scores and sexual functioning or depressive symptoms. This may be explained by the fact that, due to ongoing antipsychotic treatment, patients were in remission and—except for four who withdrew—remained so throughout the study. These findings indicate that metformin’s effects on sexual functioning and depressive symptoms cannot be attributed to baseline severity of the psychiatric disorder or to an effect of metformin on the course of schizophrenia. Similarly, differences in metformin’s effects do not appear to be related to the non–hormone-mediated actions of the antipsychotic drugs. The population we assessed differed in terms of the medications used—each of which carried a high risk of inducing hyperprolactinemia—reflecting prescribing practices in Europe, where a variety of antipsychotic drugs are commonly prescribed [42]. However, the proportions of patients receiving first- and second-generation antipsychotics were similar across groups, and antipsychotic treatment—including specific medications and doses—was comparable. Moreover, no differences were observed in baseline FSFI and BDI-II scores despite at least 12 weeks of stable antipsychotic treatment prior to the study, and dose adjustments were not permitted.

A prior investigation indicated that the detrimental effect of hyperprolactinemia on sexual function in young women is not amplified by the concomitant presence of vitamin D deficiency [8]. The current study, which included participants matched for prolactin concentrations and the extent of carbohydrate metabolism impairment while differing with respect to vitamin D status, confirms these findings. As recommended [43], vitamin D status was assessed based on serum 25OHD concentrations, which substantially minimized the potential underestimation of dietary vitamin D intake and possible absorption disturbances. Elevated prolactin levels may attenuate the negative influence of vitamin D deficiency, as evidenced by a higher risk of sexual dysfunction and lower FSFI scores in women with increased monomeric prolactin concentrations compared with women with vitamin D deficiency [4,11]. Alternative explanations include shared pathophysiological pathways contributing to sexual dysfunction in both conditions, as well as unrecognized baseline differences related to interactions between low vitamin D status and prolactin secretion and metabolism. Evidence supporting the latter hypothesis is provided by the observed associations between 25OHD levels and sexual function in women with uncorrected vitamin D deficiency or insufficiency.

The present study is the first to demonstrate that metformin exerts a beneficial effect on all domains of sexual functioning in women with hyperprolactinemia, provided that vitamin D status is normal. Nevertheless, even under these conditions, improvements in the global sexual functioning index and in individual domain scores assessed by the FSFI questionnaire were less pronounced than those observed in women treated with cabergoline [8]. This difference is most likely attributable to the substantially weaker prolactin-lowering effect of metformin compared with cabergoline, which is regarded as the most potent agent for reducing prolactin levels regardless of the underlying cause of hyperprolactinemia [44]. Thus, from a sexological standpoint, metformin is a less effective treatment than dopamine agonists (particularly cabergoline), and in the management of sexual dysfunction associated with iatrogenic hyperprolactinemia, it should be used only when these medications cannot be administered. In contrast to women with adequate vitamin D status, patients with inadequately corrected vitamin D deficiency or insufficiency exhibited improvements limited to only two FSFI domains, without a corresponding enhancement in overall sexual functioning. These findings support the assessment of 25OHD levels in all sexually active women with drug-induced hyperprolactinemia prior to initiating metformin therapy, and potentially during treatment and subsequent vitamin D repletion. Ongoing monitoring is reasonable, despite the absence of an overall drug-related effect on 25OHD levels in the study population, because of the potential for seasonal variability in vitamin D status among individual patients.

A notable limitation of this study is that it included exclusively women with coexisting prediabetes, a restriction imposed for ethical reasons. According to current guidelines, metformin is not indicated for individuals with normal insulin sensitivity, although such a population would have been ideal for examining the behavioral consequences of interactions between metformin and vitamin D status [45]. It should be emphasized, however, that hyperprolactinemia predisposes individuals to adverse alterations in carbohydrate metabolism; consequently, women with this condition frequently present with type 2 diabetes, prediabetes, or metabolic syndrome [46,47]. Given the well-established association between type 2 diabetes and sexual dysfunction [48,49], the study was limited to women with only mild impairments in glucose homeostasis. While prediabetes also appears to confer a risk for sexual dysfunction, its impact is less pronounced than that of diabetes, and differences have been documented in sexual response among women with isolated impaired fasting glucose, isolated glucose intolerance, or a combination of both conditions [50,51]. Although the specific type of prediabetes was not considered during patient selection, the study groups did not differ in this regard.

Improvements in carbohydrate metabolism may explain the observed increases in lubrication and sexual satisfaction scores across all study groups and may partially account for the more pronounced changes observed in women with normal vitamin D status compared with those with uncompensated vitamin D deficiency or insufficiency. This interpretation is supported by correlations between improvements in these domains and reductions in HbA_1c_ levels, a sensitive marker of average glucose levels over the past 2–3 months [52]. The relatively modest association between metformin’s effects on carbohydrate metabolism and sexual function, compared with our previous study, likely reflects the additional influences of hyperprolactinemia, antipsychotic treatment, schizophrenia (the primary indication for such treatment), and, in some patients, low vitamin D status—each shown to adversely affect sexual function [3,4,9,10,11,53,54]. 

Integrating behavioral observations with hormonal measurements enables the identification of the primary mechanisms underlying improvements in sexual function in metformin-treated women, which are independent of changes in carbohydrate metabolism. A decrease in prolactin levels (total and monomeric), observed exclusively in women with normal vitamin D status, together with positive correlations between the magnitude of prolactin reduction and increases in both the global FSFI score and scores across all FSFI domains, highlights the pivotal role of monomeric prolactin itself. Testosterone also appears to exert a significant influence, though it is less pronounced compared with that of prolactin. Similar to prolactin, changes in testosterone concentrations occurred solely in women with normal vitamin D status. Moreover, mediation analysis revealed that testosterone may mediate the effects of prolactin reduction on female sexual function, but its impact was found to be limited to libido and arousal. These observations are consistent with previous results from our research team [55]. We reported that reproductive-age women receiving exogenous vitamin D due to euthyroid autoimmune thyroiditis exhibited higher testosterone levels compared with those receiving alternative therapies aimed at reducing antithyroid antibody titers, and these elevated levels correlated with improvements in libido and excitement. The partial mediatory role of testosterone in the effects of metformin in women with hyperprolactinemia is further supported by the well-established importance of this hormone in regulating female sexual function, particularly with respect to desire and arousal [56,57]. In contrast, no significant associations were observed between metformin-induced improvements in sexual function and the concentrations of estradiol, LH, FSH, or DHEA-S, despite the influence of metformin on the levels of most of these hormones in women with normal vitamin D status. These findings indicate that none of these hormones is likely to modulate sexual response in the study population. Lastly, the observed effects cannot be ascribed to changes in vitamin D homeostasis, as metformin treatment did not alter circulating 25OHD levels in any of the study groups.

The investigated patients were at increased risk of experiencing depressive symptoms compared with the general population, in which the lifetime prevalence of depression is already considerable [58]. Accordingly, the relatively low mean baseline BDI-II score appears to be secondary to the beneficial effects of antipsychotic medications on mood [59]. More importantly, metformin use—unlike its effects on sexual functioning—was associated with improved BDI-II scores and a reduced proportion of patients meeting criteria for depression only among women with normal vitamin D status. To date, mood improvement associated with metformin has been observed exclusively in women with polycystic ovary syndrome, whereas only a tendency toward mood improvement has been reported in women with type 2 diabetes [28,60]. Notably, plasma 25OHD concentrations were not assessed in either of those studies. Importantly, no effect of metformin on mood was observed in an age-matched group of women with prediabetes comparable to that included in the present study [28]. Therefore, attributing the improvement in depressive symptoms observed in the current study participants to favorable effects on markers of prediabetes appears unconvincing. The presence of a correlation between changes in BDI-II scores and improvements in sexual functioning indicates a partial association between these two effects of the medication but does not allow determination of the direction of causality. Consequently, the reduction in depressive symptom severity may have been either a cause or a consequence of improved sexual functioning. Establishing causality requires robust experimental design because the suspected cause must be manipulated to observe changes in the effect while controlling for other variables. Implementing such a task is highly challenging and was not feasible within the framework of our pilot study. Nevertheless, the findings suggest that the improvement in depressive symptoms was partially secondary to enhanced sexual functioning. This interpretation is supported by the fact that—unlike individual components of the sexual response—no correlations were observed between metformin’s effects on depressive symptoms and the biochemical parameters characterizing the study population, including prolactin and 25-hydroxyvitamin D levels. However, it cannot be excluded that mood improvement (secondary to enhanced sexual functioning and other mechanisms of action of metformin) may itself contribute to further improvement in sexual functioning. The absence of mood improvement in women with low vitamin D status may reflect attenuation of metformin’s effects by deficiency of this micronutrient, which itself has antidepressant properties [61]. A potential site of interaction between vitamin D and metformin may involve brain structures implicated in affect regulation, such as the hypothalamus, hippocampus, olfactory bulbs, and frontal cortex. These regions are characterized by the presence of 1α-hydroxylase, which plays a key role in calcitriol synthesis, as well as vitamin D receptors, and they also demonstrate the capacity to accumulate substantial amounts of metformin [62,63].

One of the key findings of this study was that metformin produced similar effects on sexual function and depressive symptoms in two distinct cohorts of women with adequate vitamin D status: those who did not use vitamin D supplements and those who received supplemental vitamin D in tablet or capsule form. Despite having normal vitamin D levels, these groups differed in their patterns of vitamin D intake. Women who had previously developed vitamin D deficiency and therefore initiated supplementation exhibited lower dietary vitamin D intake, comparable to that of patients with untreated deficiency. However, when both dietary sources and supplements were considered, their overall vitamin D intake exceeded that of women with normal vitamin D status who were not taking supplements. Importantly, among women with untreated vitamin D deficiency, sexual function was associated solely with circulating 25OHD concentrations and was unrelated to vitamin D intake. This observation supports the interpretation that the recovery of metformin’s typical effects on sexual function following vitamin D supplementation results from the correction of vitamin D deficiency at the tissue level rather than from a direct interaction between metformin and supplemental vitamin D. From a clinical perspective, these intergroup comparisons indicate that normalization of vitamin D homeostasis may restore the beneficial effects of metformin on sexual functioning and mood. However, the lack of an association between sexual functioning outcomes and 25OHD concentrations within the normal range argues against the need to target a specific 25OHD threshold during supplementation.

It is essential to acknowledge other inherent limitations of the research protocol employed. Although the study was adequately powered to detect statistically significant differences between groups, the limited number of participants and the brief observation period constrain the ability to draw definitive conclusions. Owing to its cohort design, the study was susceptible to selection and attrition biases. Although the FSFI and BDI-II are well-validated instruments, their reliability—like that of other self-report measures—may have been influenced by human error, subjective bias, or intentional misreporting. It remains possible that metformin’s effects on sexual function and depressive symptoms differ in women with severe hyperprolactinemia, a population not represented in this study. Caution should be exercised when extrapolating these findings to women with hyperprolactinemia of other etiologies. Because the assessment of vitamin D intake was limited to the most commonly consumed Polish dishes to ensure the questionnaire was suitable for patients’ cognitive abilities, vitamin D intake from less commonly consumed foods may have been underestimated. The exclusion of individuals with drug-induced hyperprolactinemia accompanied by untreated severe vitamin D deficiency precludes drawing conclusions regarding the effects of metformin in this group. We cannot completely exclude the possibility of differences in health-seeking behaviors or attitudes toward medical adherence. Even with safeguards implemented at the design stage, the results may have been affected by regression to the mean, whereby extreme values tend to shift toward the average over time [64]. Lastly, the six-month study duration may be insufficient to detect long-term physiological changes in vitamin D status, particularly given year-round recruitment and seasonal fluctuations that could have masked or amplified the drug’s effects.

## 5. Conclusions

In women of reproductive age with antipsychotic-induced hyperprolactinemia and normal vitamin D status, metformin improves sexual function and mood, primarily by lowering prolactin levels and also by increasing testosterone levels and improving glucose homeostasis. Uncompensated vitamin D deficiency or insufficiency impairs the metabolic effects of metformin and abolishes its effect on elevated prolactin levels. These attenuated biochemical responses are linked to smaller improvements in sexual function compared with women with adequate vitamin D status. Normal vitamin D status is also required for metformin to exert its beneficial effects on mood, which appears to be positively linked to improved sexual functioning. These findings suggest that chronic metformin therapy may confer beneficial effects on sexual function and depressive symptoms in reproductive-age women with drug-induced hyperprolactinemia, but only when vitamin D homeostasis is maintained. The adverse effects of vitamin D deficiency or insufficiency are transient and resolve with effective supplementation. Given the novelty of these observations and the study’s inherent limitations, further research is warranted to replicate and validate these findings in larger, longitudinal cohorts.

## Figures and Tables

**Table 1:** Demographic and clinical characteristics of the study population (intention-to-treat analysis).

Variable	Group I	Group II	Group III
Number of patients	25	25	25
Age (years)	37 ± 7	36 ± 6	36 ± 7
First/second generation antipsychotic (%) *	52/48	52/48	48/52
Duration of antipsychotic treatment (months)	48 ± 18	45 ± 14	46 ± 17
Isolated impaired fasting glucose/isolated impaired glucose tolerance/impaired fasting glucose and impaired glucose tolerance (%)	40/40/20	44/40/16	44/36/20
Body mass index (kg/m^2^)	24.5 ± 4.6	24.3 ± 4.8	25.5 ± 5.0
Smokers (%)/Number of cigarettes a day (*n*)/Duration of smoking (months)	44/8 ± 5/125 ± 60	48/8 ± 6/118 ± 55	44/9 ± 6/120 ± 58
Physical activity: total/several times a week/once a week/once a month (%)	80/40/24/16	76/36/20/20	80/36/24/20
Primary or vocational/secondary/university education (%)	16/36/48	16/40/44	12/44/44
Occupational activity/Blue-collar/white-collar/pink-collar workers (%)	76/20/32/24	80/16/36/28	76/16/36/24
Number of sexual partners (*n*)	2.8 ± 1.2	2.7 ± 1.1	2.5 ± 1.0
Number of marriages (*n*)/duration of marriages (months)	1.2 ± 0.6/102 ± 46	1.3 ± 0.5/98 ± 40	1.2 ± 0.6/96 ± 42
Number of deliveries (*n*)/Number of miscarriages (*n*)	1.4 ± 0.7/0.6 ± 0.5	1.3 ± 0.7/0.6 ± 0.5	1.2 ± 0.6/0.5 ± 0.5
Systolic blood pressure (mm Hg)	124 ± 20	126 ± 19	128 ± 17
Diastolic blood pressure (mm Hg)	77 ± 5	78 ± 5	79 ± 7
Daily vitamin D intake with food (µg)	18.3 ± 7.0 ^b,c^	9.3 ± 3.5	8.9 ± 2.9
Daily vitamin D intake with food and tablets/capsules (µg)	18.3 ± 7.0 ^c^	48.4 ± 17.8 ^a,c^	8.9 ± 2.9
25OHD (nmol/L)	114.5 ± 18.8 ^b^	52.0 ± 12.5	116.1 ± 19.0 ^b^
Fasting glucose (mmol/L)	6.20 ± 0.48	6.18 ± 0.44	6.24 ± 0.42
HOMA-IR	4.0 ± 1.7	3.8 ± 1.8	4.3 ± 1.9
HBA_1c_ (%)	6.0 ± 0.3	6.0 ± 0.3	6.1 ± 0.2
Total prolactin (ng/mL)	61.3 ± 10.2	62.2 ± 11.0	59.7 ± 9.8
Monomeric prolactin (ng/mL) **	58.4 ± 12.4	59.0 ± 11.5	57.1 ± 11.8
LH (U/L)	4.12 ± 1.10	4.40 ± 1.25	4.31 ± 1.40
FSH (U/L)	4.28 ± 1.04	4.19 ± 1.08	4.42 ± 1.42
Estradiol (pmol/L)	124 ± 49	132 ± 60	118 ± 52
Testosterone (nmol/L)	0.85 ± 0.40	0.82 ± 0.42	0.91 ± 0.50
DHEA-S (µmol/L)	5.00 ± 1.25	4.85 ± 1.01	4.68 ± 1.12
PANNS-6	10.1 ± 1.9	9.8 ± 2.2	10.3 ± 2.1
FSFI	24.80 ± 4.81	25.50 ± 4.52	24.42 ± 4.01
BDI-II	15.7 ± 3.5	15.5 ± 3.0	16.1 ± 3.4

Group I: vitamin D-sufficient women not receiving vitamin D supplementation; Group II: vitamin D-sufficient women receiving vitamin D supplementation due to previously documented vitamin D deficiency or insufficiency; Group III: women with untreated vitamin D deficiency or insufficiency. Unless otherwise specified, values are reported as mean ± standard deviation. * Only medications with a documented propensity to induce hyperprolactinemia were permitted. ** Results of assays performed on samples from 18 patients per group. ^a^ *p* < 0.05 vs. group I; ^b^ *p* < 0.05 vs. group II; ^c^ *p* < 0.05 vs. group III.

**Table 2:** Average and individual daily antipsychotic doses among participants.

Drugs	Group I		Group II		Group III	
	Average Dose (mg)	Individual Doses (mg)	Average Dose (mg)	Individual Doses (mg)	Average Dose (mg)	Individual Doses (mg)
Phenothiazines	400 (promazine); 150 (perazine)	400 and 400 (promazine); 100 and 200 (perazine)	350 (promazine) 200 (perazine)	300 and 400 (promazine); 200 and 200 (perazine); 6 (perphenazine);	350 (promazine)	300 and 400 (promazine); 200 mg (perazine)
Haloperidol	3.2	2, 2, 3, 4 and 5	3.0	2, 3 and 4	3.5	2, 4, 4 and 4
Sulpiride	500	400 and 600	400	400 and 400	467	400, 400 and 600
Flupenthixole	1.5	1 and 2	1.67	1, 2 and 2	1.5	1 and 2
Risperidone	3.40	1, 2, 2, 3, 4, 4, 4, 4, 4 and 6	3.45	1, 2, 2, 2, 3, 4, 4, 4, 5, 5 and 6	3.50	1, 2, 2, 2, 3, 4, 4, 4, 4, 4, 6 and 6
Amisulpride	400	200 and 600	-	400	-	400

Group I: vitamin D-sufficient women not receiving vitamin D supplementation; Group II: vitamin D-sufficient women receiving vitamin D supplementation due to previously documented vitamin D deficiency or insufficiency; Group III: women with untreated vitamin D deficiency or insufficiency.

**Table 3:** Sexual dysfunction and depressive symptoms of varying severity in reproductive-age women with antipsychotic-induced hyperprolactinemia, stratified by vitamin D status.

Variable	Group I	Group II	Group III
**FSFI score ≤ 26.55**			
At baseline	18 (78)	18 (82)	19 (86)
At study completion	7 (30) ^a,b,c^	6 (27) ^a,b,c^	17 (77)
**Depressive symptoms**			
At baseline	15 (65)	14 (64)	16 (73)
At study completion	10 (43) ^a,b,c^	9 (41) ^a,b,c^	15 (68)
**Mild symptoms**			
At baseline	13 (56)	12 (55)	14 (64)
At study completion	9 (39) ^a,b,c^	8 (36) ^a,b,c^	13 (59)
**Moderate symptoms**			
At baseline	2 (9)	2 (9)	2 (9)
At study completion	1 (4)	1 (5)	2 (9)
**Severe symptoms**			
At baseline	0 (0)	0 (0)	0 (0)
At study completion	0 (0)	0 (0)	0 (0)

Group I: vitamin D-sufficient women not receiving vitamin D supplementation; Group II: vitamin D-sufficient women receiving vitamin D supplementation due to previously documented vitamin D deficiency or insufficiency; Group III: women with untreated vitamin D deficiency or insufficiency. Values represent the number of patients, with the percentage shown in square brackets. ^a^ *p* < 0.05 vs. the corresponding value in group III. ^b^ *p* < 0.05 vs. baseline values within the same group. ^c^ *p* < 0.05—percentage changes from baseline were more pronounced than in Group III.

**Table 4:** Correlations between the effects of metformin on biochemical variables and sexual functioning in the study population.

Correlated Variables		Group I	Group II	Group III
Δ FSFI score	Δ Total prolactin	0.43 ^b^	0.44 ^b^	0.46 ^b^
Δ FSFI score	Δ Monomeric prolactin	0.41 ^b^	0.43 ^b^	0.44 ^b^
Δ Desire	Δ Total prolactin	0.31 ^a^	0.34 ^a^	0.29 ^a^
Δ Desire	Δ Monomeric prolactin	0.29 ^a^	0.29 ^a^	0.31 ^a^
Δ Desire	Δ Testosterone	0.51 ^c^	0.49 ^c^	0.53 ^c^
Δ Arousal	Δ Total prolactin	0.35 ^a^	0.32 ^a^	0.28 ^a^
Δ Arousal	Δ Monomeric prolactin	0.34 ^a^	0.34 ^a^	0.29 ^a^
Δ Arousal	Δ Testosterone	0.47 ^b^	0.48 ^c^	0.46 ^b^
Δ Lubrication	Δ Total prolactin	0.41 ^b^	0.37 ^b^	0.43 ^b^
Δ Lubrication	Δ Monomeric prolactin	0.44 ^b^	0.39 ^b^	0.40 ^b^
Δ Lubrication	Δ HbA_1c_	0.40 ^b^	0.43 ^b^	0.29 ^a^
Δ Orgasm	Δ Total prolactin	0.51 ^c^	0.46 ^b^	0.43 ^b^
Δ Orgasm	Δ Monomeric prolactin	0.47 ^b^	0.43 ^b^	0.46 ^b^
Δ Sexual satisfaction	Δ Total prolactin	0.40 ^b^	0.37 ^b^	0.41 ^b^
Δ Sexual satisfaction	Δ Monomeric prolactin	0.43 ^b^	0.44 ^b^	0.42 ^b^
Δ Sexual satisfaction	Δ HbA_1c_	0.37 ^b^	0.44 ^b^	0.28 ^a^
Δ Pain	Δ Total prolactin	0.38 ^b^	0.41 ^b^	0.39 ^b^
Δ Pain	Δ Monomeric prolactin	0.42 ^b^	0.35 ^a^	0.41 ^b^

Group I: vitamin D-sufficient women not receiving vitamin D supplementation; Group II: vitamin D-sufficient women receiving vitamin D supplementation due to previously documented vitamin D deficiency or insufficiency; Group III: women with untreated vitamin D deficiency or insufficiency. Data are presented as correlation coefficients (r values). ^a^ *p* < 0.05, ^b^ *p* < 0.01, ^c^ *p* < 0.001.

**Table 5:** Correlations between the effects of metformin on sexual functioning and baseline 25OHD concentrations in women with untreated vitamin D deficiency or insufficiency (group III).

Δ FSFI score	25OHD	0.46 ^b^
Δ Desire	25OHD	0.50 ^c^
Δ Arousal	25OHD	0.42 ^b^
Δ Lubrication	25OHD	0.42 ^b^
Δ Orgasm	25OHD	0.35 ^a^
Δ Sexual satisfaction	25OHD	0.43 ^b^
Δ Pain	25OHD	0.36 ^b^

Data are presented as correlation coefficients (r values). ^a^ *p* < 0.05, ^b^ *p* < 0.01, ^c^ *p* < 0.001.

**Table 6:** Correlations between the effects of metformin on sexual functioning and depressive symptoms in the study population.

Correlated Variables		Group I	Group II	Group III
Δ BDI-II	Δ FSFI score	0.43 ^b^	0.44 ^b^	0.46 ^b^
Δ BDI-II	Δ Desire	0.49 ^c^	0.43 ^b^	0.48 ^c^
Δ BDI-II	Δ Arousal	0.52 ^c^	0.41 ^b^	0.40 ^b^
Δ BDI-II	Δ Lubrication	0.47 ^c^	0.39 ^b^	0.36 ^b^
Δ BDI-II	Δ Orgasm	0.42 ^b^	0.50 ^c^	0.40 ^b^
Δ BDI-II	Δ Sexual satisfaction	0.38 ^b^	0.41 ^b^	0.39 ^b^
Δ BDI-II	Δ Pain	0.46 ^b^	0.48 ^c^	0.28 ^a^

Group I: vitamin D-sufficient women not receiving vitamin D supplementation; Group II: vitamin D-sufficient women receiving vitamin D supplementation due to previously documented vitamin D deficiency or insufficiency; Group III: women with untreated vitamin D deficiency or insufficiency. Data are presented as correlation coefficients (r values). ^a^ *p* < 0.05, ^b^ *p* < 0.01, ^c^ *p* < 0.001.

## Data Availability

The data that support the findings of this study are available from the corresponding author upon reasonable request.
